# Superstructures of fluorescent cyclodextrin via click-reaction

**DOI:** 10.3762/bjoc.9.94

**Published:** 2013-04-29

**Authors:** Arkadius Maciollek, Helmut Ritter, Rainer Beckert

**Affiliations:** 1 Institute of Organic Chemistry and Macromolecular Chemistry, Heinrich-Heine-University Duesseldorf, Universitaetsstraße 1, 40225 Duesseldorf, Germany; 2 Institute of Organic Chemistry and Macromolecular Chemistry, Friedrich-Schiller-University, Humboldtstr. 10, 07743 Jena, Germany

**Keywords:** fluorescent dye, cyclodextrins, host–guest interaction, supramolecular polymer

## Abstract

Mono-(6-azido-6-deoxy)-β-cyclodextrin (CD) was covalently attached to an alkyne-modified 5-methyl-2-(pyridin-2-yl)thiazol-4-ol yielding a fluorophore containing CD in a click-type reaction. Intermolecular complexes were formed by poly(host–guest)-interactions. The supramolecular structures were characterized by ^1^H NMR-ROESY spectroscopy, dynamic light scattering, UV–vis spectroscopy, fluorescence spectroscopy, and asymmetric flow field-flow fractionation. By adding potassium adamantane-1-carboxylate*,* the thiazol dye is displaced from the CD-cavity and the elongated noncovalent polymeric structures collapse*.*

## Introduction

Most small heterocyclic molecules show low fluorescence. However, as we reported earlier, substituted 4-hydroxythiazoles have a high fluorescent nature [1–2]. These optical properties can be used for the development of sensor molecules, blue emitting dyes, luminescent materials or as donor–(π-conjugated-bridge)–acceptor-type dyes in dye-sensitized solar cells [3–5]. The etherification of 4-hydroxythiazole by using propargyl bromide opens up a wide field of modification through click chemistry with azides or thioles [6–7]. Accordingly, following our former work with mono azide modified CD, we were encouraged to combine the fluorescent hydroxythiazole dye with CD [8–10]. Such water soluble spectroscopically active hosts can be used as potentially biological markers, as molecular sensors, or for chiral recognition in aqueous solution [11–13]. Thus, in this present paper we describe the spectroscopic and structural behavior of a “CD-Click-Fluorophore” in the absence and presence of a competitive guest in water.

## Results and Discussion

**Scheme 1:**
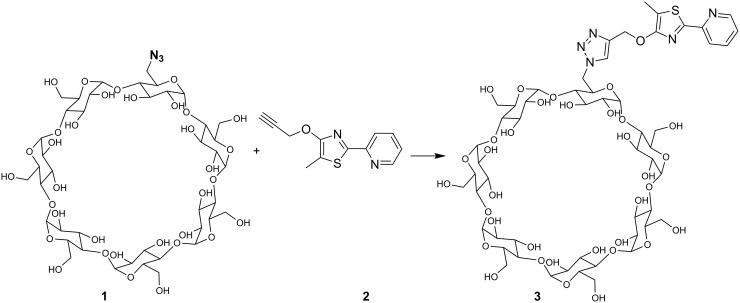
Synthesis of fluorescent cyclodextrin **3** by click-chemistry.

**Figure 1:**
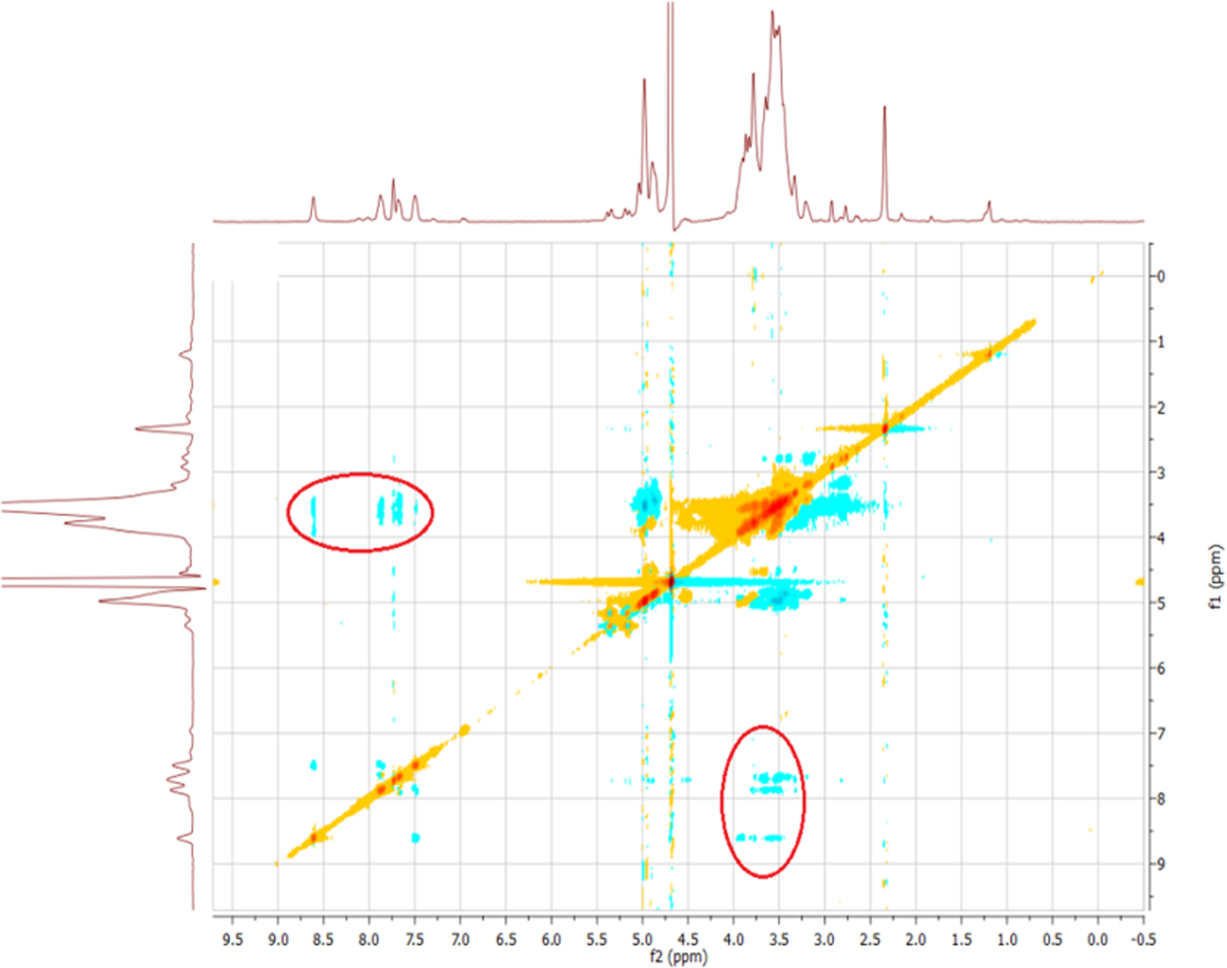
^1^H NMR-ROESY spectrum of the modified CD **3**.

The water-soluble fluorescent cyclodextrin was synthesized via copper-catalyzed cycloaddition (Scheme 1). The existence of the resulting product was confirmed by MALDI–TOF spectrometry, ^1^H NMR and IR spectroscopy. The formation of host–guest structures between the thiazole functionality and β-CD was proven by ^1^H NMR rotating-frame Overhauser effect spectroscopy (ROESY) (Figure 1). NOE correlation signals of the β-CD cavity protons between 4 and 3.5 ppm and the aromatic protons of the pyridine moiety 8.6–7.4 ppm were observed, clearly proving the formation of complexes.

However, no NOE interaction between protons of the methyl group of the thiazole and the triazole proton itself with CD is noticed. This indicates that only the inclusion of the pyridine moiety in the hydrophobic cavity of the CD takes place.

**Figure 2:**
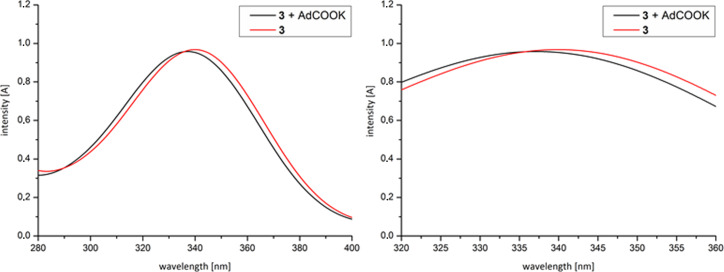
UV–vis spectrum of **3** (4 × 10^−4^ M) with and without a 10-fold excess of potassium adamantane-1-carboxylate.

The formation of supramolecular structures was also proven by UV–vis spectroscopy and fluorescence spectroscopy*.* Figure 2 shows the UV–vis absorption spectra of **3** in water with the characteristic band at λ_max_*=* 340 nm. The addition of a 10-fold excess of potassium adamantane-1-carboxylate, as competitive guest for CD leads to a hypsochromic shift with λ_max_ *=* 336 nm, through the exclusion of the fluorophore moiety from the hydrophobic cavity of CD. This exchange causes a change in the HOMO–LUMO gap [14–15].

**Figure 3:**
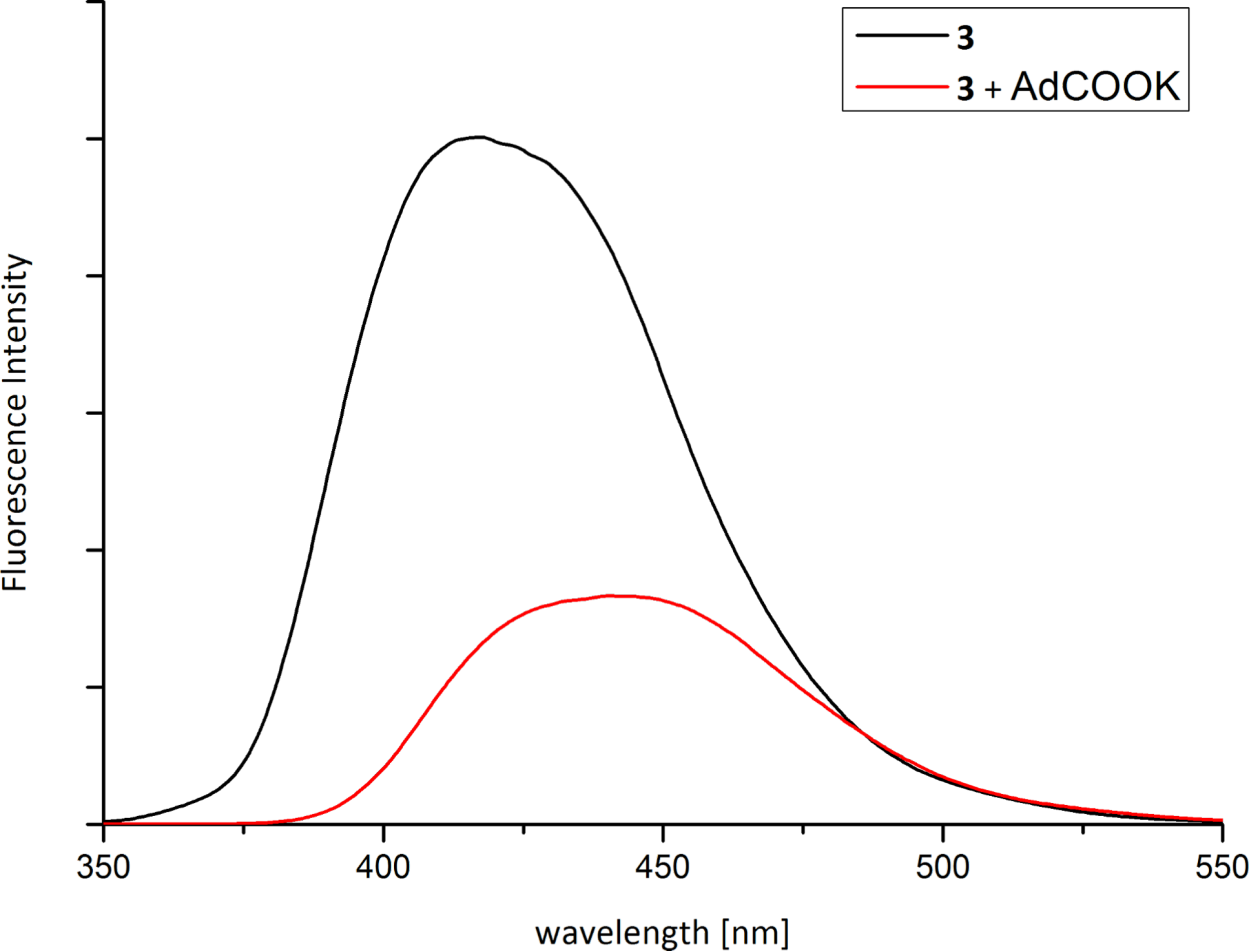
Fluorescence spectrum of **3** (4 × 10^−4^ M) with and without a 10-fold excess of 1*-*adamantanecarboxylic acid.

Figure 3 shows the fluorescence spectra of **3** in aqueous solution. Upon addition of potassium adamantane-1-carboxylate the maximum wavelength changes from 417 to 442 nm and the fluorescence intensity decreases. The inclusion of the fluorophore in the CD cavity causes an increase in the fluorescence intensity resulting from the decrease of the intramolecular rotational degrees of freedom of the molecule, compared to a solvent-accommodated fluorophore outside the cavity [12,14,16].

**Figure 4:**
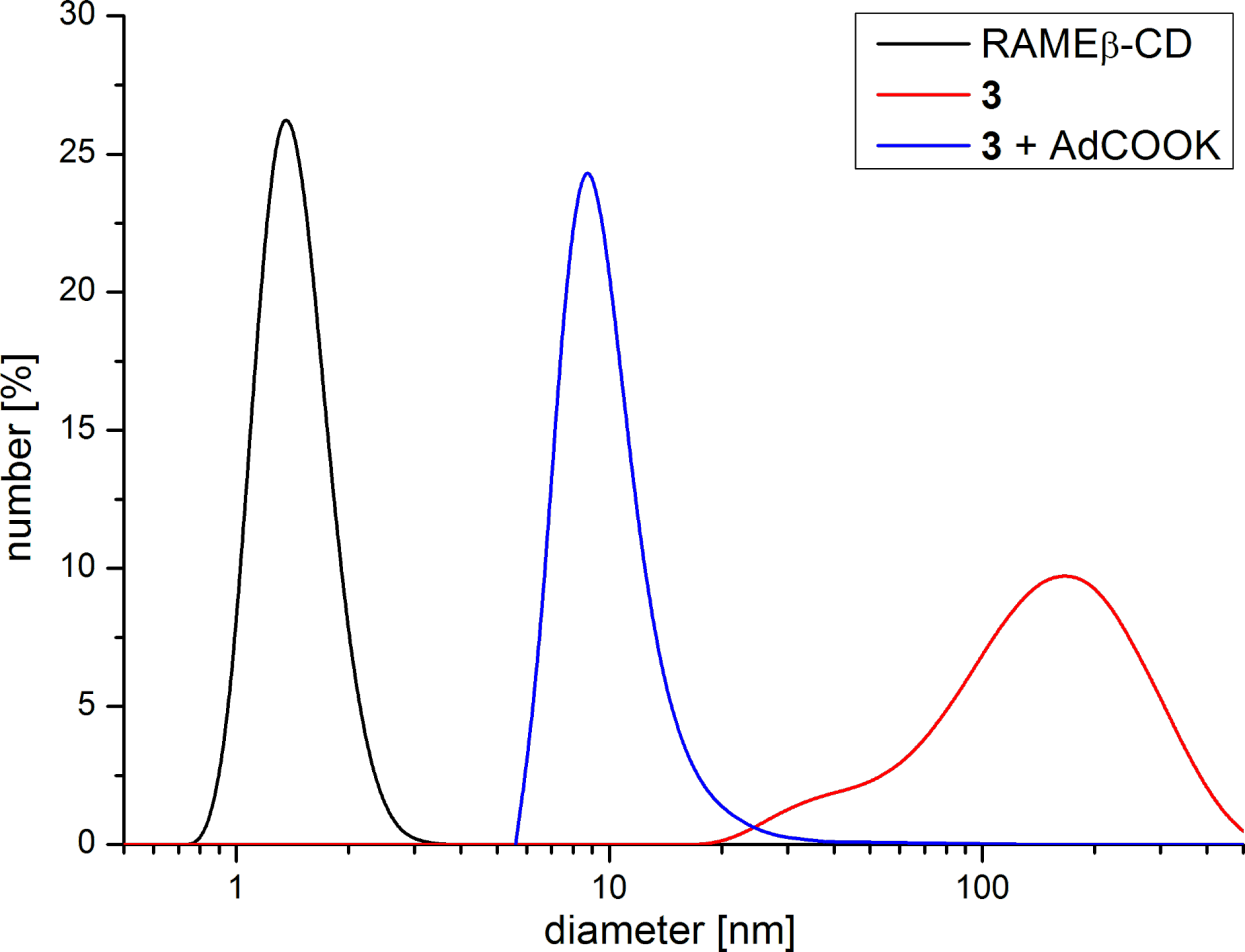
DLS measurement of **3** with and without a 10-fold excess of potassium adamantane-1-carboxylate; black: hydrodynamic diameter of randomly methylated β-CD, blue: mixture of monomer and oligomer after adding potassium adamantane-1-carboxylate, red: supramolecular polymer of **3**.

To investigate the expected intermolecular formation of supramolecular structures in aqueous solution, dynamic light scattering (DLS) experiments were performed (Figure 4). Hydrodynamic diameters up to 200 nm indicate the postulated formation of intermolecular complexes. Thus, repeating complexation of one rigid fluorophore moiety through the CD-function of the next monomer molecule takes place [17–18]. Accordingly, the formation of linear polymers via supramolecular monomer linkages can be claimed. Besides, intermolecular chain interactions may also take place due to hydrogen-bond interactions between the CD units.

**Figure 5:**
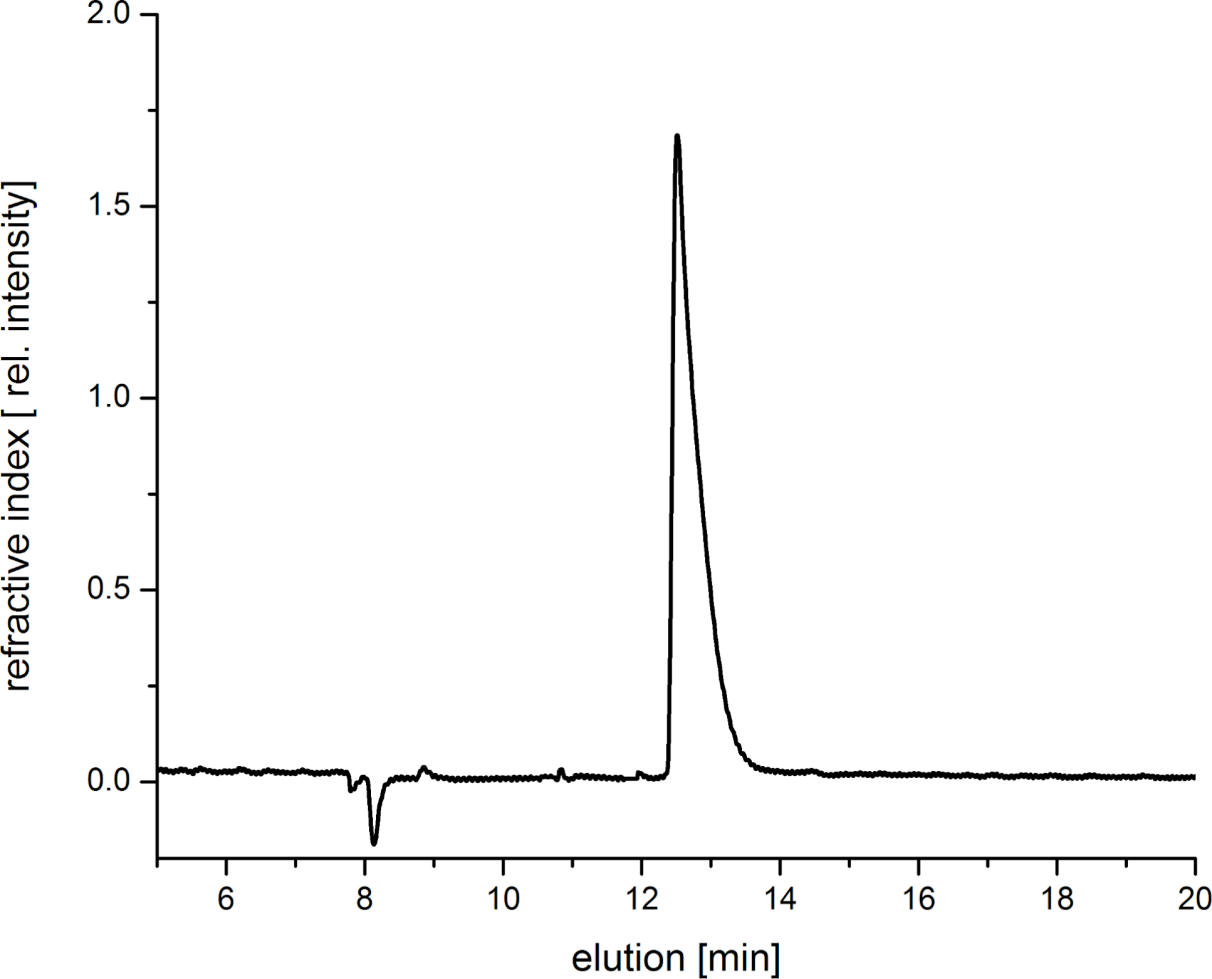
AF4 elution diagram of **3**.

By adding potassium adamantane-1-carboxylate the pyridine moiety is displaced from the cavity. As a consequence, the noncovalent structures collapse to monomeric and oligomeric CD units and the hydrodynamic diameter decreases by as much as 5 nm*.* Asymmetric flow field-flow fractionation experiments coupled with a three-angle light scattering detector confirm also the formation of intermolecular superstructures (Figure 5). Structures with a molar mass of *M*_n_ up to 100,000 g/mol and also a hydrodynamic diameter up to 200 nm were detected. The conformation plot, i.e., the plot of the log of the radius as a function of the log of the molar mass, with a value of 0.7 estimates an elongated shape of the noncovalent structures. Upon addition of potassium adamantane-1-carboxylate no signals of the polymeric structure were detected.

## Conclusion

In summary, we have presented the synthesis of a fluorescent cyclodextrin via click reaction. The changes in the spectroscopic properties of the fluorescent cycloadduct were investigated in the presence and absence of the competitive guest potassium adamantane-1-carboxylate. The intermolecular formation of polymeric structures with elongated shape, through poly(host–guest)-interactions, was found. Furthermore, we were able to show the collapse of the supramolecular polymers upon addition of potassium adamantane-1-carboxylate.

## Experimental

### General remarks

All reagents used were commercially available (Sigma-Aldrich, Acros Organics) and used without further purification. β-CD was obtained from Wacker Chemie GmbH, Burghausen, Germany and was used after drying overnight with a vacuum oil pump over P_4_O_10_. *N,N*-Dimethylformamide (DMF) was purchased from Fluka, Germany. Dimethyl sulfoxide-*d*_6_ (99.9 atom % D) and deuterium oxide, D_2_O, were obtained from Deutero GmbH, Germany. ^1^H NMR spectra were recorded on a Bruker Avance DRX 300 at 20 °C, shifts (δ) are given relative to signals arising from the solvent.

FT IR spectra were recorded on a Nicolet 6700 FT IR spectrometer equipped with an ATR unit. Matrix-assisted laser desorption/ionization-time-of-flight mass spectrometry (MALDI–TOFMS) was performed on a Bruker Ultraflex TOF mass spectrometer. Ions were formed with a pulsed nitrogen laser (25 Hz, 337 nm) and the molecular masses were recorded in linear mode. 2,5-Dihydroxybenzoic acid (DBH) in acetonitrile/water was used as a matrix.

Mass spectrometric experiments (MS) were performed on a Thermo Finnigan Trace DSQ (Dual-Stage Quadrupole) mass spectrometer. Ionization was carried out by electron ionization (EI). The absorption spectra were measured on a Specord 210 Plus UV–visible spectrophotometer. Fluorescence spectra were recorded on a Perkin Elmer LS55 luminescence spectrometer. AF4 measurements in ultrapure water were carried out on a combined system comprising the following elements: refractive index detector Optilabrex (Wyatt Technologies, laser wavelength 658 nm), three angle light scattering detector miniDawn TREOS (Wyatt Technologies, laser wavelength 658 nm, detector angles at 43.5°, 90.0° and 136.5°), UV detector Waters 486 (Waters), pump, degasser and autosampler (Agilent 1200, Agilent technologies). The molecular weight was calculated with Astra5 software from static light scattering data, by using the Zimm model. As concentration source, the refractive index was used. Calibration of the system was performed with bovine serum albumin. Dynamic light scattering (DLS) experiments were carried out with a Malvern Zetasizer Nano; ZS ZEN 3600 at a temperature of 20 °C. The particle size distribution is derived from a deconvolution of the measured intensity autocorrelation function of the sample by a General Purpose Method (non-negative least squares) algorithm included in the DTS software. Microwave-assisted synthesis was performed using a CEM Discover Synthesis Unit (monomode system). The temperature was measured by infrared detection maintained at a constant value by power modulation. Reactions were performed in closed vessels.

**Synthesis of mono-(6-azido-6-deoxy)-β-CD (1):** Mono-(6-azido-6-desoxy)-β-cyclodextrin was synthesized according to the known procedure [8].

**Synthesis of 2-[5-methyl-4-(prop-2-yn-1-yloxy)-1,3-thiazol-2-yl]pyridine (2):** The alkyne modified thiazol was synthesized according to the literature procedure [2].

**Synthesis of fluorescent β-cyclodextrin 3:** 1.3 g (1.15 mmol) of mono-(6-azido-6-desoxy)-β-cyclodextrin (**1**), 396 mg (1.73 mmol) 2-[5-methyl-4-(prop-2-yn-1-yloxy)-1,3-thiazol-2-yl]pyridine (**2**), 29 mg (147 µmol) sodium ascorbate and 18 mg (73.5 µmol) copper(II) sulfate pentahydrate were suspended in 5 mL dimethylformamide in a pressure-resistant microwave test tube provided with a magnetic stirring bar. The tube was sealed and placed in the microwave and irradiated at 140 °C and 100 W for 30 min. The product was precipitated in cold acetone, filtered and washed three times with acetone.

^1^H NMR (300 MHz, DMSO-*d*_6_, rt) δ 8.57 (br, 1H, ArH), 8.16–8.01 (br, 2H, CH, ArH), 7.98–7.86 (br, 1H, ArH), 7.47–7.38 (br, 1H, ArH), 5.79–5.68 (br, 14H, OH), 5.40 (br, 2H, CH_2_), 4.89–4.79 (br, 7H, CH), 4.51–4.44 (br, 6H, OH), 3.75–3.53 (br, 28H, CH), 2.22 (br, 3H, CH_3_) ppm; IR: 3301 (OH), 2923 (C-H), 1653 (C=C), 1548 (ring vibr.), 1329 (OH), 1153 (COH), 1078 (COC), 1027 (COH) cm^−1^; MS (MALDI–TOF) (acetonitrile/water 1:10): *m*/*z* = 1412 [M + Na]^+^.
